# Knowledge of Vitamin D among First-year Medical Undergraduate Students of a Medical College: A Descriptive Cross-sectional Study

**DOI:** 10.31729/jnma.6196

**Published:** 2021-03-31

**Authors:** Pratibha Manandhar, Naresh Manandhar, Sunil Kumar Joshi

**Affiliations:** 1 Department of Community Medicine, Kathmandu Medical College Teaching Hospital, Duwakot, Bhaktapur, Nepal

**Keywords:** *knowledge*, *medical students*, *vitamin D*

## Abstract

**Introduction:**

Vitamin D also known as the sunshine vitamin, helps in bone metabolism and calcium homeostasis. It is estimated that one billion people in the world have vitamin D deficiency making it a public health problem. The objective of this study is to find out the knowledge regarding vitamin D among first-year medical undergraduate students of a medical college.

**Methods:**

This is a descriptive cross-sectional study carried out from 2nd February 2020 to 15th February 2020 at Kathmandu Medical College Teaching Hospital, Duwakot among medical undergraduate students of a medical college. Ethical clearance was received from the Institutional Review Committee of Kathmandu Medical College Teaching Hospital (Reference Number: 2812201809). A structured self-designed multiple-choice questionnaire on vitamin D knowledge was used. Convenient sampling method was applied and statistical analysis was done with Statistical Package for the Social Sciences 20 version.

**Results:**

Out of total 157 undergraduate medical students, 21 (13.3%) exhibited good knowledge, 116 (73.9.8%) had average, and 20 (12.8%) had poor knowledge. There were 83 (52.9%) Bachelor of Medicine and Bachelor of Surgery students, 39 (24.8%) were Bachelor of Dental Surgery and 35 (22.3%) were Nursing students.

**Conclusions:**

We found a satisfactory level of knowledge of vitamin D among first-year undergraduate medical students of a medical college when compared to similar studies. The majority of students from all three disciplines had average knowledge of vitamin D.

## INTRODUCTION

Vitamin D is a fat-soluble, sunshine hormone needed during infancy, adolescence, adulthood, and pregnancy. It is important for normal calcium absorption from the gut and bone growth.^1,2^ Sunlight is the best source of vitamin D and its presence in food is limited.^3^ Cutaneous synthesis of vitamin D is obtained by the conversion of 7-dehydrocholesterol to Cholecalciferol (Vitamin D3) by ultraviolet radiation from the sun.^4^

A common health problem like cardiovascular diseases, type 1 diabetes mellitus, periodontal disease, hypertension, and many cancers is due to vitamin D deficiency or insufficiency.^5^ A poor level of knowledge and an inadequate level of awareness are two of the main risk factors for vitamin D deficiency.^6^

Various studies on vitamin D among students has shown a certain gaps of knowledge and its perception regarding its importance.^7^ As they are future health providers, it will help for the progression of health-promoting behaviors for themselves and the community.^8^ The main objective of this study is to find out the knowledge regarding vitamin D among first-year medical undergraduate students of medical college.

## METHODS

This is a descriptive cross-sectional study conducted among first-year students who were just enrolled in the 23rd batch Bachelor of Medicine and Bachelor of Surgery (MBBS), 8th batch Bachelor in Dental surgery (BDS) and 15th batch Nursing at Kathmandu Medical College Teaching Hospital (KMC), Duwakot, Bhaktapur, Nepal. Ethical approval was obtained from the Institutional Review Committee of Kathmandu Medical College Teaching Hospital (Reference Number .2812201809) and written consent was signed by all participants. Students who consented to participating in the study and who were attending regular classes were included. The study period was from 2nd February 2020 to 15th February 2020. The convenient sampling method was applied. The sample size was calculated as,

$$\begin{array}{ll} {\text{n}}_{\circ } & ={\text{Z}}^{\text{2}}\times \text{p}\times \text{q}/{\text{e}}^{\text{2}}\\ & ={\left(1.96\right)}^{2}\times (0.5)\times (1-0.5)/{\left(0.05\right)}^{\text{2}}\\ & =384.16\\ & =385 \end{array}$$

Now, adjusting the sample size in the finite population,

$$\begin{array}{ll} \text{n} & ={\text{n}}_{\circ }/1+[({\text{n}}_{\circ }-1)/\text{N}]\\ & =385/1+[(384-1)/185]\\ & =125.04\\ & =126 \end{array}$$

Where,

n_∘_ = required sample size
n = adjusted sample size
Z = 1.96 at 95% Confidence Interval
p = prevalence, 50%
q = 1-p
e = margin of error, 8%

The calculated sample size was 126. Taking a 20% non-response rate, the final sample size was 152. But we collected data from 157 students using a structured questionnaire. It consisted of section A about socio-demographic characteristics. Section B which was a structured self-designed multiple-choice questionnaire consists of 51 marks for the score of knowledge regarding vitamin D (ever heard the word vitamin D, the main and food source of Vitamin D, the daily recommended dosage of Vitamin D, the average time exposure under ultraviolet radiation, deficiency-related disease, the dosage of Vitamin D). The total knowledge score of each participant was calculated and categorized as good, average, and poor. Out of the total 51 scores, poor knowledge was defined as 0-11, the average was 12-21 and good was >22 respectively.

The data were analyzed using the International Business Machine Statistical Package of Social Sciences (SPSS) version 20.0. Point estimate at 95% of Confidence Interval along with frequency and proportion for binary was analyzed.

## RESULTS

Out of total of 157 undergraduate medical students, 21 (13.3%) exhibited good knowledge, 116 (73.9.8%) had average, and 20 (12.8%) had poor knowledge.

Among the 157 students, 83 (52.9%) were MBBS, 39 (24.8%) were BDS, and 35 (22.3%) were Nursing students. Among MBBS students, 57 (68.7%) were boys, 57 (71.1%) were Nepalese and 60 (72.3%) were non-vegetarian. Similarly, among the BDS students, 33 (84.5%) were girls, 34 (87.21%) were non-vegetarian and all of them were Nepalese. So, out of 35 Nursing students, all were girls, 30 (85.7%) were non-vegetarian and all were Nepalese. Fifty eight (69.9%) MBBS students, 28 (71.8%) BDS, and 34 (97.1%) Nursing students are of less than 20 yrs (Table 1).

**Table 1:** Demographics of the participant undergraduate medical students.

Variables	MBBS n (%)	BDS n (%)	Nursing n (%)
Gender			
Boys	57 (68.7)	6 (15.4)	-
Girls	26 (31.3)	33 (84.6)	35 (100)
Citizens			
Nepali	59 (71.1)	39 (100)	35 (100)
Indian	23 (27.7)	-	-
Maldivian	1 (1.2)	-	-
Age groups			
<20 yrs	58 (69.9)	28 (71.8)	34 (97.1)
20- 25 yrs	25 (30.1)	11 (28.2)	1 (2.9)
Food Preference			
Non-Vegetarian	60 (72.3)	34 (87.2)	30 (85.7)
Vegetarian	16 (19.3)	3 (7.7)	3(8.6)
Eggtarian	7 (8.4)	2 (5.1)	2 (5.7)

Based on the response given by 157 students, 21 (13.7%) exhibited good knowledge, 116 (73.9%) had average and 20 (12.8%) had poor knowledge. Among the 83 MBBS students, 11 (13.3%) were good, 64 (77.1%) were average and 8 (9.6%) was in the poor category. Similarly, out of 39 BDS students, 7 (17.9%) were good, 26 (66.7%) were average and 6 (15.4%) were in the poor category. Whereas out of 35 nursing students, 3 (8.5%) were in a good category, 26 (74.3%) average and 6 (17.1%) were in the poor category (Table 2).

**Table 2:** Knowledge of Vitamin D among first-year undergraduate medical students.

Category	MBBS n (%)	BDS n (%)	Nursing n (%)	Total n (%)
Poor	8 (9.6)	6 (15.4)	6 (17.1)	20 (12.8)
Average	64 (77.1)	26 (66.7)	26 (74.3)	116 (73.9)
Good	11 (13.3)	7 (17.9)	3 (8.5)	21 (13.7)
Total	83 (100)	39 (100)	35 (100)	157 (100 )

## DISCUSSION

Among 157 undergraduate medical students, 21 (13.3%) exhibited good knowledge, 116 (73.9.8%) had average, and 20 (12.8%) had poor knowledge. This study found an average knowledge regarding Vitamin D among first-year medical undergraduates of MBBS, BDS, and Nursing disciplines. In Asian countries, Vitamin D deficiency may be overlooked as in the perception that vitamin D deficiency is unlikely to occur in these regions which receives sunshine throughout the year.^8-10^ There is very limited awareness campaigns for the importance of vitamin D among general populations and high-risk populations.^11^ The health benefits of Vitamin D are beyond bone health confirmed from the various studies. Recently, Vitamin D received enormous attention worldwide and its deficiency has become pandemic.^12^

A study done by Amri F Al, et al. in Saudi Arabia showed out of 186 participants, 95 (51.3%) had good knowledge regarding Vitamin D and 90 (48.7%) had poor knowledge.^13^ Similarly, a study done by Juanid R, et al. in Rawalpindi Medical University, Pakistan found that among 340 medical students, 183 (54%) students had good knowledge, 145 (42.6%) had average knowledge and 12 (3.5%) students had poor knowledge.^14^ Whereas our study had highlighted a satisfactory level i.e. 116 (73.9%) students had average knowledge of Vitamin D. The possible reason behind this could be that these students are from 10+2 science background and have just enrolled in first-year course and classes on Vitamin D has not been started yet.

A study was done by Lhamo Y, et al. in Vardhman Mahavir Medical College, New Delhi, India showed that there is gaps in basic knowledge about vitamin D among medical undergraduate students.^12^ Even in our study, most of the students have average knowledge regarding vitamin D, which could be accepted as being first-year medical students and their knowledge will increase at the final year during the study period. These research findings have reinforced the need of sensitization regarding vitamin D among medical undergraduates. Awareness about vitamin D at this stage of the medical profession would benefit their health and increase health-related behavior change as being future medical practitioners.^15^

This study was conducted in a limited sample size in a single institution. The study's participants were selected using a convenient sampling technique. Therefore, the results may not be generalized. Future studies done in multiple institutions employing random sampling techniques should be done.

## CONCLUSIONS

Findings obtained via this study depict a satisfactory level of knowledge of vitamin D among undergraduate MBBS, BDS and nursing students when comapred to similar studies. Good knowledge of the disease could mean that the students may develop sound clinical knowledge in subsequent years of training.
